# Is there added risk in resurfacing a femoral head with cysts?

**DOI:** 10.1186/1749-799X-6-55

**Published:** 2011-10-17

**Authors:** Thomas P Gross, Fei Liu

**Affiliations:** 1 Midlands Orthopaedics, P.A. Columbia, South Carolina, USA

## Abstract

**Background:**

Femoral head cysts have been identified as a risk factor for early femoral failures after metal-on-metal hip resurfacing arthroplasty (HRA) based on limited scientific data. However, we routinely performed HRA if less than 1/3 of the femoral head appeared destroyed by cysts on the preoperative radiograph. This study was undertaken to analyze whether there was an added risk of early femoral failures in HRA when femoral head cysts were present.

**Methods:**

This retrospective case-control study included 939 MOM HRAs operated by a single surgeon with use of the posterior minimally invasive surgical (MIS) approach between November 2005 and January 2009. Patients with all diagnoses except osteonecrosis were included. Among them, 117 HRAs had femoral head cysts ≥ 1 cm identified in surgery. All cysts were treated with bone grafting using acetabular reamings packed into the cavitary defect (instead of filling the cysts with cement). The control group, which had no cyst observed at the time of surgery, was randomly selected from our database using computer algorithms to match those cases in the study group for the parameters of surgical date, age, gender, body mass index, diagnosis, femoral fixation method, and the size of the femoral component.

**Results:**

The minimum follow-up was 24 months for both groups. The early femoral failure rate in the study group was 3/117 (2.6%) and 0/117 in the control group; there was no statistical difference between these two groups (*P *= 0.08). In the study group, there were two femoral neck fractures (revised): both occurred in patients having a cyst size of 1 cm^3^; and there was one femoral component loosening at 3-year follow up in a patient having a cyst size of 2 cm^3^.

**Conclusion:**

Although the risk of early femoral failures among the group with cysts appeared higher than the group without cysts, we could not demonstrate a significant statistical difference between the two groups. It is possible that bone grafting cysts rather than cementing them may account for the low failure rate, and that this technique may minimize the risk of resurfacing a femoral head with cysts.

## Background

Hip resurfacing arthroplasty (HRA) with metal-on-metal bearings has become an established and viable hip arthroplasty option for the younger patient with higher activity levels due to bone preservation. This technique may also make revision surgery less complicated [1,2]. In Europe, the rate of resurfacing has varied between 6% and 9% with 6% in France, 9% in Germany, and 7% in the UK [1,3]. In Australia, the hip resurfacing accounts for 7.9% of all hip arthroplasty procedures. In some countries, hip resurfacing has been utilized in up to 50% of all hip arthroplasties in patients younger than 55 with a low revision rate of 2.8% at five-year follow-up post-operatively [4,5].

The risk factors for stemmed total hip arthroplasty (THA) appear to be different than for HRA^5^, and many experts have advocated that HRA may be more advisable in certain subsets of patients with severe degenerative arthritis of the hip. Risk factors have been proposed that increase the risk for HRA [6-8]. Femoral head cysts are widely believed to increase the chances of early femoral failure in HRA; however, the only scientific data that exist now to support this idea is mainly from Beaule's study [6,9,10]. In their study, femoral head cysts were identified as a risk factor for early femoral failure after metal-on-metal HRA as a part of the proposed Surface Arthroplasty Risk Index (SARI) [6]. Cysts were found to be a significant risk factor (*P *= 0.028) for early femoral failure. Our concern is that the technique of managing cysts may be important in achieving a good outcome. In Beaule's study, cysts were filled with cement; our technique is to instead fill them with acetabular reamings prior to cementation or uncemented fixation.

We were not convinced that cysts affected the failure rate provided that they involved less than one third of the prepared femoral head and that they were bone grafted instead of being filled with cement. Because the scientific evidence to support cysts as an independent risk factor was limited, we have routinely used this approach. After many years of experience with these cases, we have now undertaken this study to independently analyze what the added risk of early femoral failure in HRA was when femoral head cysts were present and treated with bone grafting. Our hypothesis in this retrospective case-control study was that femoral heads with cysts involving less than 1/3 of the prepared femoral head did not significantly affect the early femoral failure rate after HRA.

## Methods

Institutional review board (IRB) was approved for this study. From November 2005 to January 2009, the senior author (T.P.G) performed 939 metal-on-metal HRAs in 831 patients with various primary diagnoses. We excluded only the cases with osteonecrosis (ON) from the entire group because we were unable to quantify the amount of dead bone present in the prepared femoral head in such cases. Our technique for ON cases was to only remove loose dead bone and drill the well-fixed dead bone. Therefore, this technique of treating ON cases did not allow for quantification of the amount of non-viable bone. Both our study and Beaule's study analyzed the effect of femoral cysts on the early femoral failure rate before 3 years. There were no data available to determine whether cysts may affect the long-term femoral loosening rate. The posterior minimally invasive surgical (MIS) approach with the Biomet RecapTM and MagnumTM hip resurfacing system (Biomet, Warsaw, IN, USA) was used in all cases. In the first 437 cases, a cemented femoral component was used, then 502 fully porous femoral prostheses were employed. The study group consisted of 117 HRAs that had femoral head cysts (size range: 1 to 4 cm^3^) identified in surgery. A control group was selected from our database using computer algorithms to match for the parameters of surgical date, age, gender, body mass index (BMI), diagnosis, and the size and fixation technique of the femoral component. Beginning in July 2006, Dual Energy X-ray Absorptiometry (DEXA) was utilized to determine the bone mineral density of patients and recorded as a T-score. Therefore, T-score data were not available for all patients in this study. The control group included 117 HRAs that had no cyst identified at the time of surgery. There were no statistical differences between the study and the control group other than the presence or absence of femoral head cysts. All data on demographics, risk factors, surgical details, and hospital stay are listed in Table 1.

**Table 1:** Demographic and diagnosis comparison between the groups with or without cysts.

	Study Group -- with Cyst	Control Group -- Without Cyst	P-Value
**Surgical Date**	11/2005 to 1/2009	8/2005 to 12/2008	--
**Number of hips**	117	117	--
**Number of patients**	115	115	--
**Age at surgery (years)**	53 ± 6 (range: 35 to 69)	53 ± 5 (range: 34 to 65)	0.66
**Weight (lbs)**	189 ± 40 (range: 110 to 290)	186 ± 37 (range: 110 to 275)	0.5
**Body mass index**	27 ± 4 (range: 19 to 39)	27 ± 4 (range: 20 to 39)	0.59
T-score (Bone mineral density)*	0 ± 1 (range: -2.5 to 3.3)	0 ± 1 (range: -2.4 to 3.5)	0.96
**Gender**			1
Women	33 (29%)	34 (30%)	--
Men	82 (71%)	81 (70%)	--
**Side**			1
Left	53 (45%)	53 (45%)	--
Right	64 (55%)	64 (55%)	--
**Diagnosis**			0.7
Osteoarthritis	95 (81%)	95 (81%)	--
Dysplasia	20 (17%)	21 (18%)	--
Post Trauma	1 (1%)	1 (1%)	--
Others	1 (1%)	0 (3%)	--

* Not available for all the patients.

Details of the MIS surgical procedure were described in a previous study [11]. In all cases, when cysts greater than 1 cm^3 ^were present, they were thoroughly debrided and grafted with acetabular reamings and platelet concentrate. In the earlier cases in this series, the following cement technique was used. A 5-mm trough was placed on the posterior and inferior femoral head for cement escape. A thin cement mantle was applied to the femoral head (including over the bone graft) and to the undersurface of the component. The component was then impacted, expressing excess cement. No stems were cemented. In the later uncemented cases, the femoral component was simply impacted over the femoral head with an interference fit. The average total volume of the cysts in the study group was 1.8 ± 0.8 cm^3 ^(range: 1 to 4 cm^3^) (Table 2 &3). Femoral heads where total cyst volume was smaller than 1 cm^3 ^were not counted as having significant cysts. The cell saver was used in 17 cases with the average amount of 120 ± 56 cc (range: 30 to 220 cc) in the study group and was used in 16 cases with the average amount of 132 ± 52 cc (range: 30 to 220 cc) in the study group (*P *= 0.85). No blood transfusion was required in any case. Other surgical details are specified in Table 3.

**Table 2:** The information of the cyst size among the study group.

**Size of Cyst (cm**^**3**^**)**	Number	Percentage
1	52	44%
2	44	38%
3	15	13%
4	6	5%

**Table 3:** Summary of the Surgical Information between the groups with or without cysts.

	Study Group -- with Cyst	Control Group -- Without Cyst	P-Value
ASA*	2 ± 1 (range: 1 to 3)	2 ± 1 (range: 1 to 3)	0.33
**Hospital stay (days)**	2 ± 1 (range: 1 to 5)	2 ± 1 (range: 1 to 7)	0.22
**Operation time (min)**	120 ± 23 (range: 85 to 242)	109 ± 17 (range: 80 to 168)	0.004
**Size of cyst (cm**^**3**^**)**	1.8 ± 0.8 (range: 1 to 4)	0	**<0.001**
**Femoral component size (mm)**	51 ± 4 (range: 44 to 62)	51 ± 4 (range: 44 to 60)	0.78
**Fixation of femoral component**			1
**Cemented**	64	64	--
**Fully porous coated (Uncemented)**	53	53	--

* American Society of Anesthesiologists (ASA) scores.

**Figure 1:**
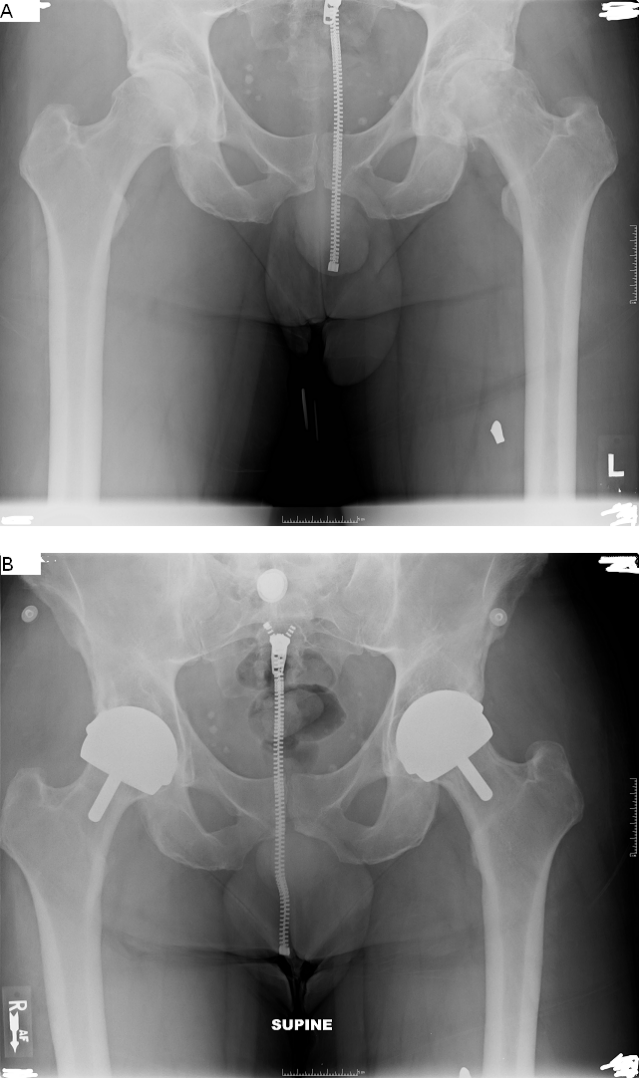
**Bilateral HRAs, male 43 years old age, the cyst size of 3 cm^3 ^on the left side and the cyst size of 0 cm^3 ^on the right side**; HHS 97 at both 3-year follow-up (left) and 1-year follow-up (right), primary diagnosis of OA for both side. A: pre-operative x-ray, B: latest post-operative xray.

Routine postoperative follow-ups were requested at six weeks, one year, two years, and every other year afterward. Harris hip score (HHS), UCLA activity score, and visual analogue scale (VAS) pain score were evaluated at every follow-up visit. Complications and failures were recorded. Anteroposterior and lateral radiographies were obtained at each follow-up (Figure 1). Radiolucencies, osteolysis, migration, reactive femoral lines, focal femoral neck narrowing, and heterotopic bone according to the Brooker scale[12] were evaluated.

The level of significance was set as 0.05 (α = 0.05) for all comparison tests in this study. The paired *t *tests were performed to compare the numeric variables between pre-operative and post-operative visits. The standard *t *tests were performed to compare the differences between numeric variables of the study and control groups. *Chi*-square tests were performed to evaluate the difference of categorical variables between these two groups. The *Kaplan-Meier *curves were used to analyze the survivorship rates using revision of femoral components as the end point among these two groups. The *Chi*-square tests were performed to approximate the results of the Wilcoxon tests in order to compare the differences of survivorship functions between groups. The null hypotheses of all of these tests were that the survivorship functions were the same between the two compared groups [13]. Also, the Pearson Chi-square tests were utilized to compare the differences of failure rates between groups without considering the time variable.

## Results

**Figure 2:**
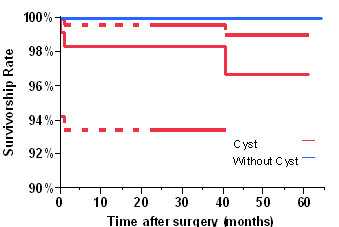
**Kaplan Meier Survivorship Curves of the group with cyst and the group without cyst after metal-on-metal HRA with 95% confidence interval using femoral component failures as the end point (P = 0.09)**.

All patients in this study had a minimum follow-up of 24 months (Table 4). No patients died in the study group. Two patients died of causes unrelated to the hip surgery after two years in the control group. Both of them were included in this study. At the latest follow-up visits, there were three femoral failures (two in men and one in a woman) in the study group; there was no femoral failure in the control group (*P *= 0.08): two cases (1.7%) were revised due to femoral neck fracture prior to six months post-operatively; one (0.9%) was revised due to femoral component loosening (presumably due to osteonecrosis). Detailed information is listed in Table 5. The survivorship curves using revision of the femoral component as an endpoint are plotted in figure 2. At 60 months postoperatively, the survivorship rates of the femoral components were 97.4% in the study group and 100% in the control group. However, there was no significantly statistical difference of failure rates between these two groups without considering the time variable (*P *= 0.08) and there was no significantly statistical difference of survivorship functions between them (*P *= 0.09). In the cyst group, there was one femoral neck fracture among 53 uncemented femoral components; and there was one femoral neck fracture and one femoral component loosening that occurred among 64 cemented femoral components. There was no significantly statistical difference of the early femoral component failures between the fixation of femoral components (*P *= 0.67).

**Table 4:** Summary of clinical outcomes between the groups with or without cysts.

	Study Group -- with Cyst	Control Group -- Without Cyst	P-Value
**Period of follow-up (months)**	42 ± 11 (range: 24 to 61)	45 ± 12 (range: 24 to 65)	0.08
**Pre-operative information**			
**HHS score**	54 ± 12 (range: 24 to 91)	55 ± 13 (range: 21 to 83)	0.2
**Post-operative information**			
**HHS score**	97 ± 6 (range: 68 to 100)	95 ± 8 (range: 71 to 100)	0.22
**UCLA score**	8 ± 2 (range: 4 to 10)	8 ± 2 (range: 3 to 10)	0.95
**VAS score in the regular day**	0 ± 1 (range: 0 to 4)	0 ± 1 (range: 0 to 4)	0.59
**VAS score in the worst day**	1 ± 2 (range: 0 to 8)	1 ± 2 (range: 0 to 7)	0.27
**Femoral radiolucency**	0 (0%)	0 (0%)	1
**Number of femoral failures (revisions)**	3 (3%)	0 (0%)	0.08
**Deceased**	0 (0%)	2 (1.7%)	0.16

**Table 5:** Detailed information of early femoral component failures in the group with cysts.

Time after surgery (Months)	**Cyst size (cm**^**3**^**)**	Femoral size (mm)	Primary diagnosis	BMI	Gender	Age	Reason of failure	Treatment of failure
0	1	48	Dysplasia	23	Female	49	Femoral Neck Fracture	Femur Revised
1	1	52	OA	35	Male	59	Femoral Neck Fracture	Femur Revised
37	2	58	OA	27	Male	53	Femoral Component Loosening	Femur Revised

Excluding the revised cases, the average post-operative HHS scores at the latest follow-up visit was 97 ± 6 in the study group and 95 ± 8 in the control group; both were improved significantly from the average pre-operative HHS scores, respectively (P < 0.001) (Table 4). There were no significant differences in the UCLA activity and VAS pain scores on the regular or worst days. Radiological analysis revealed that no hip showed evidence of femoral radiolucency or migration.

## Discussion

When comparing HRA to stemmed THA, the spectrum of complications is different. Considering that multiple bearing options are currently available for stemmed THA, the comparison between HRA and stemmed THA becomes even more difficult. Two complications that are unique to HRA are femoral neck fractures and postoperative femoral head osteonecrosis. We have therefore decided to focus on these. In combination, they represent early femoral component failures after HRA. Proximal femoral bone preservation in young active patients is the primary reason that metal-on-metal HRA was developed. However, if the risks of early femoral failures are particularly high in a certain group of patients, they may be considered poor candidates for HRA. If the alternative risks of amputating the femoral head and neck to perform a stemmed THA are much lower in this group, the theoretical advantage of bone preservation with HRA in younger patients may no longer be worthwhile. Numerous studies have focused on delineating risk factors for HSR to help the surgeon decide which patients may have too high a risk with HRA to make proximal femoral bone preservation worthwhile [6,10,14,15]. Unfortunately, it is not always clear exactly why a certain risk factor is problematic. Does a smaller component size lead to more problems because of a small area of femoral fixation^5^? Or is the problem with smaller components primarily because of more adverse wear problems [10,16,17]. The present retrospective case-control study was specifically undertaken to assess one proposed risk factor for early femoral failures: Does the presence of femoral head cysts increase the risk of early femoral failure?

Cysts in the femoral head are areas where bone loss has occurred due to the arthritic process. Therefore, it is generally believed by experts that femoral head cysts negatively impact the success rate of HRA [6,9,10]. However, to our knowledge, only few papers have reported scientific evidence that femoral head cysts are a risk factor for HRA ^5^. Because it seems logical that cysts might affect femoral fixation, this belief has largely gone unchallenged, despite the fact that the evidence available is limited. Beaule et al ^5 ^proposed a SARI on the basis of a study of 92 HRAs done in patients under 40 years of age. The average follow-up was 3 years (range: 2-5.6 years). Survivorship with revision for early femoral failure as an endpoint was 97% (two femoral neck fractures, one femoral loosening). There were two additional radiographically loose femoral components (migration) and eight additional possibly loose femoral components (complete stem radiolucency). This formed the problematic group (N = 13). A univariate analysis of multiple risk factors was done. Points were assigned to certain risk factors based on their odds ratio in this analysis. Two points were assigned for cysts > 1 cm^3^, 2 points for weight under 82 kg, one point for UCLA Activity score above 6, and one point for previous hip surgery. The maximum score was 6. The SARI was found to be significantly higher in the 13 problematic hips than in the remainder of the hips in the series (*P *< 0.001). Femoral head cysts were found in 53% of well-functioning hips while they were present in 92% of problematic hips (*P *= 0.028). Their data implicate the presence of femoral head cysts (>1 cm^3^) as a risk factor for HRA. It does not quantify the added risk for failure due to cysts. Also, the cysts in Beaule's study were managed by debridement and filling with cement.

Our study contradicts these findings (Table 6). Our study was based on approximately twice as many patients (117 with cysts in the study group and 117 in the matched control group). The follow-up was similar. The revision rate for early femoral failure was slightly less and there were no radiographically loose components in our study. Our study group of 117 patients was compared to a control group that was computer matched for factors that have been proposed as risk factors for early femoral failure (see Table 1 &3). In addition, UCLA activity scores and incomplete data on bone mineral density showed no differences between the two groups. We could not demonstrate a statistically significant difference in the rate of early femoral failures when cysts ≥ 1 cm^3 ^were present in the femoral head. Our data did indicate that the extra operative time required in managing the cysts using our technique required on average 11 minutes (*P *= 0.004). There are several possible explanations for this fact. Firstly, although our study had more power than the comparison study, it is still possible that a Type 2 error is present. It is possible that the presence of femoral head cysts is a weak negative factor, which our study was not adequately powered to pick up. But, if this is not the case, the presence of femoral head cysts should not be a weak risk factor that should not affect the surgeons' decision-making process. Secondly, our management of cysts was different than that of Dr. Amstutz in the comparison study [6]. We fill our cysts with acetabular reamings rather than cement. This may have positively affected the outcome of our cases with cysts to the point where no difference could be found in comparison to cases without cysts. In another study, Beaule has presented evidence that filling femoral head cysts with cement can significantly increase the temperature within the femoral head. This may lead to more devascularization and a higher rate of complications if the cement filling technique is used. In the present study, we could not find the difference in the failure rate based on the method of femoral fixation chosen. Furthermore, our previous comparative study has shown no difference in the early failure rate of HRA performed with hybrid or uncemented fixation [18]. In Beaule's study, there were 3% revisions due to early femoral failure, but also 2% radiographically loose components, and 8.7% possibly loose components for a total of 14% "problematic hips". We had only 1.5% total problematic hips in our study. But the studies are not directly comparable. Our patients were older (which may increase the complication rate) and did not include the diagnosis of ON (which may decrease the complication rate).

**Table 6:** Comparison of the results between Beaule & Amstutz's study and the present study.

	**Beaule & Amstutz **[6]	Gross & Liu
**Publish year**	2004	2011
**# of patient**	92	234
**# with cysts > 1 cm**^**3**^	54	117
**# without cysts**	38	117
**Follow-up length (yrs)**	3 (range: 2 to 5.6)	3.5 yr (range: 2 to 5.4)
**UCLA activity score**	7.1	8
**Femoral revision rate**	3%	1.3%
**Femoral migration**	2%	0%
**Femoral radiological loosening**	8.7%	0%
**P value of femoral component failures between cyst and non-cyst group**	**0.028**	0.08

## Conclusions

In summary, our study, with a control group matched for other previously proposed risk factors for early femoral loosening, could not demonstrate that femoral head cysts were an independent negative risk factor for failure of the femoral resurfacing component. However, we caution that this may be due to the way we treat femoral head cysts with bone grafting, rather than filling them with cement. We therefore recommend that the presence of cysts within the femoral head, as long as they comprise less than 1/3 of the remaining prepared femoral head, be eliminated as a risk factor for HRA. We suggest that other surgeons consider bone grafting cysts rather than filling them with cement. Comparison studies to further compare these two techniques would be valuable.

## Competing interests

The authors wish to disclose that Thomas P. Gross receives the royalty from Biomet.

## Authors' contributions

TPG designed this study, collected the data, and drafted the manuscript. FL designed this study, analyzed the data, performed statistical analyses and drafted the manuscript. All of the authors read and approved the final version of this study.
